# Bad to the bone: *Candida (Candidozyma) auris* vertebral osteomyelitis treated with combination antifungal therapy followed by a novel long-acting echinocandin

**DOI:** 10.1128/asmcr.00114-25

**Published:** 2025-10-07

**Authors:** Lauryn B. Jenkins, Robbie L. Christian, Boris A. Karaman, Mahmoud A. Ghannoum, Khalid M. Dousa

**Affiliations:** 1 Department of Medicine, Louis Stokes Cleveland VA Medical Center, Cleveland, Ohio, USA; 2 Department of Radiology, Louis Stokes Cleveland VA Medical Center, Cleveland, Ohio, USA; 3 Case Western Reserve University School of Medicine, Cleveland, Ohio, USA; 4 University Hospitals Cleveland Medical Center, Cleveland, Ohio, USA; Rush University Medical Center, Chicago, Illinois, USA

**Keywords:** *Candida auris*, echinocandins, amphotericin, osteomyelitis

## Abstract

**Background:**

*Candida auris* is an emerging multidrug-resistant yeast associated with healthcare-associated infections and high mortality. Vertebral osteomyelitis due to *Candida auris* is rare and challenging to treat due to limited data on antifungal bone penetration, prolonged treatment duration, and resistance to multiple antifungal classes. Long-acting agents such as rezafungin may offer promising outpatient options, though clinical experience remains limited.

**Case Summary:**

A 70-year-old male developed vertebral osteomyelitis/discitis at T3–T4 due to *Candida auris*, following multiple catheter-related bloodstream infections and *C. auris* candidemia. Initial treatment included dual antifungal therapy with liposomal amphotericin B and micafungin, selected based on *in vitro* susceptibility and preclinical synergy data. Therapy was complicated by severe electrolyte disturbances, requiring early discontinuation of amphotericin B. He transitioned to rezafungin and completed nearly 3 months of treatment at home, contributing to a total of 6 months of antifungal therapy in alignment with IDSA guidelines. Rezafungin was generally well tolerated, with only mild hypokalemia and episodic migraine-like symptoms. The patient achieved complete clinical recovery with the resolution of symptoms and normalization of inflammatory markers. No relapse was reported at the 6-month follow-up.

**Conclusion:**

This case highlights the complexity of managing invasive *Candida auris* osteomyelitis and underscores the utility of dual antifungal combination therapy to enhance efficacy and potentially prevent the development of resistance during the intensive phase of treatment. It also demonstrates the feasibility of using rezafungin as an option for long-term outpatient management. Given the limited clinical experience with combination therapy and rezafungin use, further data are needed to inform standardized treatment approaches.

## INTRODUCTION

*Candida auris* is an emerging multidrug-resistant yeast increasingly recognized in invasive infections, including vertebral osteomyelitis (1). Treatment often requires prolonged antifungal therapy—frequently exceeding several months—which can be complicated by drug resistance, limited bone penetration, and intolerance to available agents (2). We present a case successfully managed with combination antifungal therapy followed by step-down treatment, illustrating both therapeutic challenges and strategies to improve tolerability and treatment completion.

## CASE PRESENTATION

A 70-year-old male with a history of goblet cell adenocarcinoma of the appendix underwent a right hemicolectomy at an outside facility. His postoperative course was complicated by an enterotomy, necessitating small bowel resection and placement of a Hickman catheter for total parenteral nutrition. Approximately 4 months later, he developed recurrent catheter-related bloodstream infections (CRBSIs), resulting in serious complications including L3-L4 osteomyelitis/discitis due to *Corynebacterium* spp., *Staphylococcus lugdunensis* septic arthritis of the knee, and bacteremia with carbapenem-resistant *Acinetobacter baumannii*. Each episode was attributed to the central indwelling line and was successfully managed with targeted antimicrobial therapy, line removal, and eventual replacement. Seven months after his initial surgery, the patient developed a polymicrobial CRBSI caused by extended-spectrum beta-lactamase (ESBL)-producing *Klebsiella pneumoniae* and *Candida auris*. In the setting of new-onset back pain, spinal MRI revealed T3–T4 vertebral osteomyelitis/discitis. The fungal susceptibility test was performed on a blood specimen at the Cleveland Clinic’s Robert J. Tomsich Pathology and Laboratory Medicine Institute. The isolate was identified as *Candida auris*, and susceptibility testing was performed using minimal inhibitory concentration (MIC) methodology. The MIC values and interpretations were as follows: amphotericin B (1 µg/mL, susceptible), anidulafungin (0.12 µg/mL, susceptible), caspofungin (0.06 µg/mL, susceptible), micafungin (0.06 µg/mL, susceptible), and fluconazole (>256 µg/mL, resistant). Interpretation of MIC values suggests that this *Candida auris* isolate is susceptible to amphotericin B and all tested echinocandins (anidulafungin, caspofungin, and micafungin). Notably, the isolate demonstrates high-level resistance to fluconazole, consistent with global trends for *Candida auris*, which is often intrinsically resistant to azoles. Guided by this drug susceptibility, the patient received a 7-week course of intravenous ertapenem (1 g daily) and micafungin (100 mg daily), with marked clinical improvement. No follow-up imaging was performed, and intravenous antimicrobial therapy was discontinued. The central indwelling catheter was removed after subsequent bowel reconstruction surgery.

**Fig 1:**
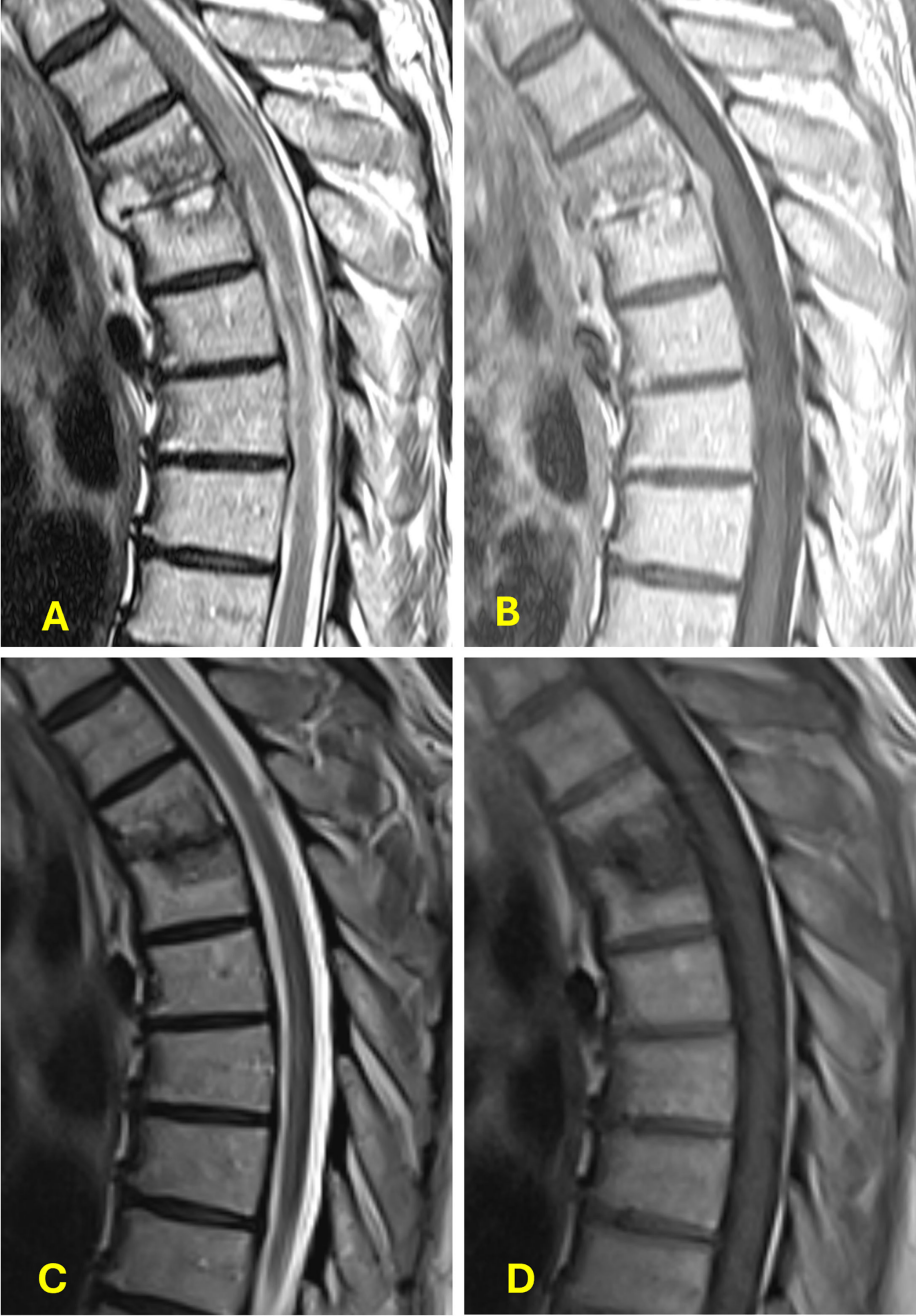
(**A**) T2-weighted image shows fluid signal in vertebral endplates and disc space, as well as anterior paraspinal. An intermediate density epidural soft tissue anterior to the cord without intrinsic fluid signal represents a phlegmon. (**B**) Contrast-enhanced T1-weighted image shows homogeneous enhancement of the anterior epidural phlegmon as well as patchy abnormal enhancement of the disc space and endplates. (**C**) T2-weighted image shows complete resolution of the anterior epidural phlegmon and resolution of abnormal fluid signal in vertebral endplates, disc space, and in the anterior paraspinal tissue. (**D**) An unenhanced T1-weighted image shows the resolution of the anterior epidural soft tissue.

The patient presented to our institution for the first time 2 months after completing antifungal therapy, with complaints of recurrence of the mid-back pain for 4 days. He was afebrile and hemodynamically stable. Physical examination revealed severe point tenderness over the lower thoracic and lumbar spine without focal neurologic deficits; lower extremity strength was preserved. Laboratory studies demonstrated a white blood cell count of 7.3 × 10⁹/L (reference range 4,000–11,000 cells per microliter [cells/µL]), elevated C-reactive protein (CRP) of 0.78 mg/L (reference range 0.00–0.50 mg/dL), and erythrocyte sedimentation rate (ESR) of 59 mm/h (reference range 0–20 mm/h). CT imaging revealed vertebral irregularities at T3–T4, and subsequent MRI revealed acute discitis/osteomyelitis. T2-weighted images showed paraspinal, vertebral endplate and disc fluid signal, and epidural soft tissue anterior to the cord. Contrast-enhanced T1-weighted images showed homogeneous enhancement of the anterior epidural soft tissue, indicating a phlegmon, as well as patchy abnormal enhancement of the disc space and endplates. These MRI findings defined acute discitis/osteomyelitis with an anterior epidural phlegmon (Fig. 1A and B). Neurosurgery was consulted, but no surgical intervention was recommended due to the lesion’s location and absence of neurologic compromise. Image-guided aspiration of the T3–T4 disc space yielded 6 mL of purulent, serosanguinous fluid, which grew *Candida auris*.

**Fig 2:**
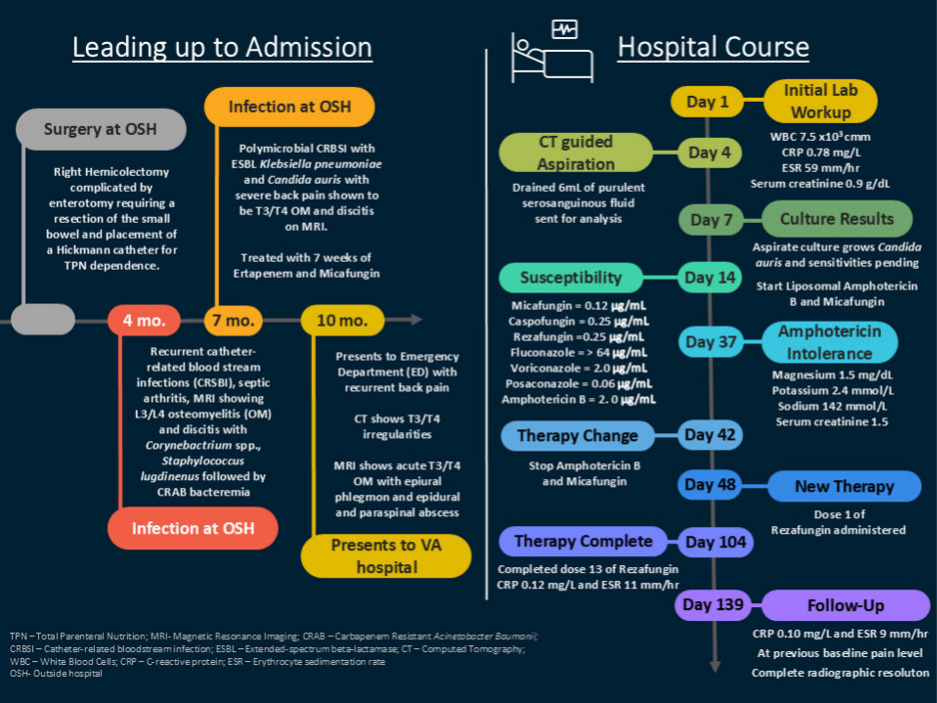
Timeline of events from admission to therapy outcome.

In susceptibility testing performed at the Mayo Clinic Microbiology laboratory for the new *Candida auris* isolate, based on Clinical and Laboratory Standards Institute (CLSI) guidelines, we observed the following MIC results: micafungin 0.12 µg/mL (susceptible), caspofungin 0.25 µg/mL (susceptible), rezafungin 0.25 µg/mL (susceptible), fluconazole >64 µg/mL (resistant), voriconazole 2 µg/mL (no interpretation available), posaconazole 0.06 µg/mL (no interpretation available), and amphotericin B 2 µg/mL (resistant) (Fig. 2). The CLSI broth microdilution method was used as it is the standard reference for antifungal susceptibility testing of *Candida auris* to azole antifungals, micafungin, and amphotericin B. Antifungal susceptibility testing for the initial blood culture isolate and the subsequent *Candida auris* isolate recovered from the T3–T4 disc space was performed according to the CLSI M27 reference method, a standardized broth microdilution approach, widely employed in research and clinical laboratories to determine MICs of antifungal agents against yeasts (3).

Empiric micafungin therapy (100 mg daily) was started immediately upon the result of the culture, and 7 days later, liposomal amphotericin B (5 mg/kg/day) was added and continued upon confirmation of susceptibilities, given preclinical reports suggesting synergistic activity between these agents. There are no published clinical data on synergy testing between micafungin and amphotericin B specifically for *Candida auris*, and we did not perform synergy testing for this isolate. Our approach was based on published pre-clinical data indicating that for *Candida* species, synergy between micafungin and amphotericin B was observed in 20%–50% of isolates, depending on the endpoint criteria used, with no antagonism detected (4, 5). During therapy, serial monitoring demonstrated improvement in inflammatory markers, with CRP declining to 0.56 mg/L and ESR to 21 mm/h by day 30 of therapy. However, by day 41, the patient developed significant electrolyte disturbances, including refractory hypomagnesemia and hypokalemia, prompting discontinuation of liposomal amphotericin B despite stable renal function.

In preparation for outpatient parenteral antifungal therapy (OPAT), he was transitioned to rezafungin: 400 mg on day 1, followed by 200 mg on day 8 and 200 mg weekly thereafter. He was discharged from a skilled nursing facility and continued rezafungin treatment at home. Aside from intermittent migraine-like episodes, he tolerated the therapy well and completed a nearly 6-month course of antifungal treatment consistent with Infectious Diseases Society of America (IDSA) guidelines for *Candida* vertebral osteomyelitis (2). After 5 months of therapy, the patient’s ESR and CRP values were 9 mm/h and 0.1 mg/dl, respectively. End of therapy follow-up T2-weighted MRI images demonstrated resolution of paraspinal, vertebral endplate, and disc fluid signal. The previously seen anterior epidural soft tissue anterior to the cord was resolved. The unenhanced T1-weighted images show no anterior epidural soft tissues (Fig. 1C and D). He was last seen in the Infectious Diseases clinic 55 days after completing therapy, at which time he was asymptomatic. At his subsequent follow-up with his primary care physician, 172 days after the termination of rezafungin therapy, he continued to report no back pain and remained asymptomatic.

Herein, we report a case of invasive *Candida auris* vertebral osteomyelitis successfully treated with a novel approach, including combination antifungal therapy followed by once-weekly monotherapy with the long-acting echinocandin rezafungin for 6 months.

## DISCUSSION

*Candida* (Candidozyma) *auris* is an emerging, multidrug-resistant yeast that has become a significant cause of healthcare-associated outbreaks worldwide. *Candida auris* can demonstrate resistance across three major classes of antifungal medications: azoles (especially fluconazole), polyenes (notably amphotericin B), and echinocandins (6). Since its first identification in 2009, *Candida auris* has demonstrated a remarkable ability to persist on skin and in the healthcare environment, leading to difficult-to-control nosocomial transmission, complicating management and infection control efforts (6). In our case, the patient was not known to be previously colonized, and there were no reports of an outbreak at the hospital he was at prior to his transfer to our hospital, nor at our hospital.

*Candida auris* is genetically diverse and is divided into at least six clades with distinct geographic distributions and antifungal resistance profiles (6). In the United States, including Ohio, the South Asian clade (Clade I) and the South American clade (Clade IV) are the predominant clades in circulation (7, 8). Clade I is associated with high rates of fluconazole resistance, while Clade IV also demonstrates significant resistance to azoles and, to a lesser extent, echinocandins (1). Sporadic cases of the South African clade (Clade III) and rare detection of the East Asian clade (Clade II) have also been reported in the United States, but these are not the main drivers of outbreaks in Ohio or nationally. The ongoing spread and clade distribution in Ohio reflect broader U.S. trends, with local transmission now the primary mode of spread rather than importation from abroad.

Between July 22, 2023, and July 22, 2025, Ohio reported a total of 33,384 cases of *Candida auris*, including both clinical infections and screening detections. The highest number of cases was reported in Franklin County, with 4,232 cases, followed by Cuyahoga County with 3,044 cases. Other counties with significant case counts include Montgomery (2,017), Hamilton (1,975), Summit (1,444), and Stark (1,199). These six counties alone accounted for over 40% of all reported cases in the state during this 2-year period, underscoring the concentration of *Candida auris* activity in Ohio’s most densely populated and healthcare-heavy regions (9).

Treatment of *Candida auris* infections poses significant challenges due to the potential for misidentification and its unpredictable resistance profile. The isolate in this case demonstrated high-level resistance to fluconazole and reduced susceptibility to amphotericin B, consistent with prior surveillance studies (10, 11). Dual antifungal therapy with liposomal amphotericin B and micafungin was initiated based on literature-reported *in vitro* synergy studies and preclinical data (4, 5). Combination antifungal therapy led to marked clinical and biochemical improvement but was complicated by severe electrolyte disturbances—particularly refractory hypokalemia and hypomagnesemia—requiring discontinuation of liposomal amphotericin B. Figure 2 summarizes the patient’s clinical course, the antifungals used, and the susceptibility results of the *Candida auris* isolate over time.

Adding another layer of complexity to therapeutic decision-making, in this case, the management of bone involvement is particularly challenging due to suboptimal drug penetration and the limited tolerability of long-term regimens, which often extend over several months (2). Moreover, there are minimal published reports of bone and joint infections caused by *Candida auris*, and data on antifungal penetration into bone tissue remain scarce, with much of it derived from animal models (12). Notably, only voriconazole, deoxycholate amphotericin B, and amphotericin B lipid complex have been shown to achieve bone concentrations well above plasma levels, while itraconazole, anidulafungin, and liposomal amphotericin B demonstrate variable penetration, reaching concentrations at or below plasma levels (13). We selected echinocandins over voriconazole based on the IDSA and CDC recommendations that echinocandins be used as first-line treatment for most *Candida auris* infections in individuals aged 2 months and older. Additionally, the decision was influenced by the need to avoid toxicity and the drug interactions commonly associated with voriconazole (2, 6). In addition, current guidelines recommend treating osteomyelitis caused by *Candida* spp. for 6–12 months, which further complicates management due to challenges with patient tolerability of long-term antifungal therapy (2, 12). Adverse effects such as QTc prolongation, nephrotoxicity, hepatotoxicity, electrolyte disturbances, drug interactions, and infusion reactions can significantly limit treatment options (2). Among antifungals, amphotericin B is associated with the most challenging adverse effect profile, although lipid formulations are generally better tolerated than the conventional formulation (2). Amphotericin B can cause intolerable toxicities, including infusion reactions, nephrotoxicity, and severe electrolyte imbalances (2). This is exemplified in the present case, where amphotericin B had to be discontinued after nearly 6 weeks of therapy due to persistent and refractory electrolyte derangements.

Rezafungin is among the most recently approved antifungal agents, a long-acting echinocandin. While rezafungin is not first in class, chemical modifications that reduce degradation allow for improved tissue penetration and prolonged half-life for once-weekly dosing (14). Chiurlo et al. successfully used rezafungin for 4 months in consolidation treatment of *Candida glabrata* prosthetic joint infection with no recurrence or adverse events (15). Additionally, Adeel and colleagues report a case of multidrug-resistant mediastinitis with *Candida glabrata* in which the patient was maintained for over a year on rezafungin with no recurrence of infection and a transient mild rash (16).

The pharmacodynamics of amphotericin B were optimized in this case using the liposomal formulation (L-AMB), which offers prolonged tissue exposure and a longer duration of action with consecutive dosing compared to conventional amphotericin B deoxycholate (17). Despite the reported resistance to drug susceptibility testing, a high-dose strategy was employed in an attempt to overcome the elevated MIC observed (17, 18). Alternative agents—particularly azole antifungals—were deemed suboptimal due to the susceptibility profile, limited bone penetration, serious concern about drug interactions, and the potential for acquired resistance, supporting the continuation of L-AMB therapy. Recent pharmacokinetic/pharmacodynamic (PK/PD) studies specifically evaluating L-AMB against *Candida auris* have demonstrated that increasing the dose to 5 mg/kg daily improves the likelihood of achieving pharmacodynamic targets for isolates with MICs up to 2 mg/L. *In vitro* PK/PD modeling suggests that for *Candida auris* isolates with a CLSI MIC of 2 mg/L, 5 mg/kg of L-AMB is required to achieve stasis; however, isolates with MICs > 2 mg/L are unlikely to be treatable, even with dose escalation (17, 18).

Due to the necessity for prolonged therapy, the patient was transitioned to rezafungin. While clinical data on rezafungin are still limited, particularly regarding its efficacy against invasive candidiasis caused by *Candida auris*, its favorable pharmacokinetics and tolerability profile suggest it could be a promising option for outpatient antifungal management (14–16, 19). In this case, rezafungin was generally well tolerated, aside from episodic migraine-like symptoms and mild hypokalemia. The patient received rezafungin for nearly 3 months, contributing to a total of 6 months of antifungal therapy in accordance with IDSA guidelines for *Candida* osteomyelitis (2). Hypokalemia developed after the third dose (serum potassium 2.8 and 2.4 mmol/L; reference range 3.5–5.0 mmol/L) and was effectively managed with oral potassium supplementation (20 mEq BID). The patient had a history of migraines, previously controlled with intermittent triptan use. Following the third rezafungin dose, he experienced a migraine within 30 minutes and was prescribed rizatriptan 10 mg. Similar episodes occurred after subsequent doses and were managed with rizatriptan and ibuprofen. While a temporal association was observed, it remains unclear whether rezafungin directly triggered the migraines or if other confounders contributed. This potential adverse effect was reported to the pharmaceutical manufacturer (Melinta Therapeutics).

### Conclusion

This case underscores several key considerations in the management of *Candida auris* osteomyelitis: the importance of early suspicion and targeted diagnosis, the role of combination antifungal therapy in overcoming resistance, and the potential utility of novel agents such as rezafungin for long-term outpatient therapy. Furthermore, it highlights the need for a multidisciplinary approach, incorporating infectious diseases, radiology, interventional procedures, and close outpatient follow-up. Given the rarity of this clinical presentation, further research and case accumulation are needed to inform standardized treatment protocols for invasive *Candida auris* infections beyond candidemia.
